# [4-(Dimethyl­amino)­phen­yl]diphenyl­phosphine selenide

**DOI:** 10.1107/S1600536812042602

**Published:** 2012-10-20

**Authors:** Wade L. Davis, Alfred Muller

**Affiliations:** a Research Centre for Synthesis and Catalysis, Department of Chemistry, University of Johannesburg (APK Campus), PO Box 524, Auckland Park, Johannesburg, 2006, South Africa

## Abstract

In the title compound, C_20_H_20_NPSe, the P atom lies in a distorted tetra­hedral environment. The Tolman cone angle is 157° indicating steric crowding at this atom. In the crystal, weak C—H⋯Se inter­actions create linked dimeric units and C—H⋯π inter­actions are also observed.

## Related literature

For investigations into the steric and electronic properties of phospho­rus containing ligands, see: Roodt *et al.* (2003 ▶); Otto & Roodt (2004 ▶); Muller *et al.* (2008 ▶); Cowley & Damasco (1971 ▶); Allen & Taylor (1982 ▶); Allen *et al.* (1985 ▶). For the free phosphine related to the title compound, see: Dreissig & Plieth (1972 ▶). For the oxide analogue of the title compound, see: Lynch *et al.* (2003 ▶). For the related phosphine selenide, see: Phasha *et al.* (2012 ▶). For cone angles, see: Tolman (1977 ▶); Otto (2001 ▶). For details on the conformational fit of mol­ecules using *Mercury*, see: Macrae *et al.* (2006 ▶); Weng *et al.* (2008*a* ▶,*b* ▶). For a description of the Cambridge Structural Database, see: Allen (2002 ▶). For background on Bent’s rule, see: Bent (1961 ▶).
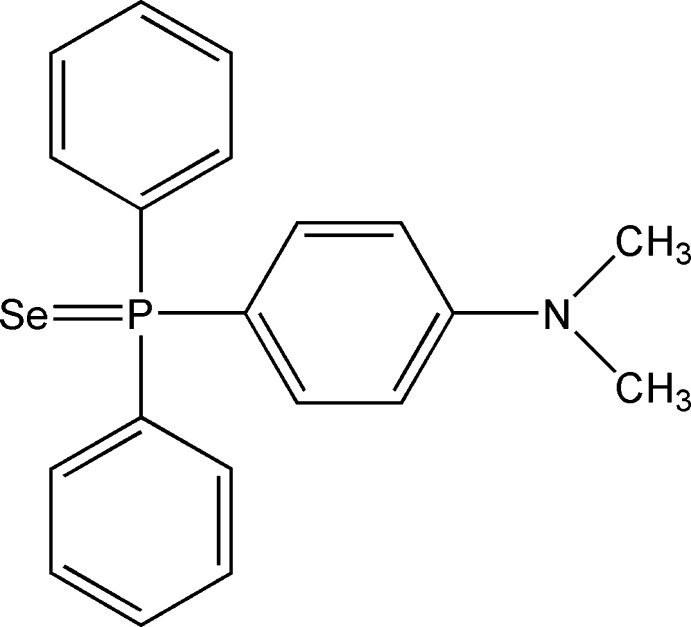


## Experimental

### 

#### Crystal data

C_20_H_20_NPSe
*M* *_r_* = 384.3
Monoclinic, 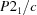

*a* = 12.1757 (13) Å
*b* = 10.6173 (11) Å
*c* = 17.5211 (14) Å
β = 128.098 (5)°
*V* = 1782.5 (3) Å^3^
*Z* = 4
Mo *K*α radiation
μ = 2.20 mm^−1^
*T* = 100 K
0.22 × 0.11 × 0.09 mm

#### Data collection

Bruker APEX DUO 4K CCD diffractometer
Absorption correction: multi-scan (*SADABS*; Bruker, 2008 ▶) *T*_min_ = 0.643, *T*_max_ = 0.827
31924 measured reflections
4553 independent reflections
3798 reflections with *I* > 2σ(*I*)
*R*_int_ = 0.050

#### Refinement

*R*[*F* ^2^ > 2σ(*F* ^2^)] = 0.036
*wR*(*F* ^2^) = 0.097
*S* = 1.06
4553 reflections
210 parameters
H-atom parameters constrained
Δρ_max_ = 0.53 e Å^−3^
Δρ_min_ = −0.72 e Å^−3^

### 

Data collection: *APEX2* (Bruker, 2011 ▶); cell refinement: *SAINT* (Bruker, 2008 ▶); data reduction: *SAINT* and *XPREP* (Bruker, 2008 ▶); program(s) used to solve structure: *SIR97* (Altomare *et al.*, 1999 ▶); program(s) used to refine structure: *SHELXL97* (Sheldrick, 2008 ▶); molecular graphics: *DIAMOND* (Brandenburg & Putz, 2005 ▶); software used to prepare material for publication: *publCIF* (Westrip, 2010 ▶) & *WinGX* (Farrugia, 1999 ▶).

## Supplementary Material

Crystal structure: contains datablock(s) global, I. DOI: 10.1107/S1600536812042602/yk2075sup1.cif

Structure factors: contains datablock(s) I. DOI: 10.1107/S1600536812042602/yk2075Isup2.hkl

Supplementary material file. DOI: 10.1107/S1600536812042602/yk2075Isup3.cml

Additional supplementary materials:  crystallographic information; 3D view; checkCIF report

## Figures and Tables

**Table 1:** Hydrogen-bond geometry (Å, °) C*g*1 and C*g*2 refer to the centroids of the C7–C12 and C13–C18 rings, respectively.

*D*—H⋯*A*	*D*—H	H⋯*A*	*D*⋯*A*	*D*—H⋯*A*
C20—H20*A*⋯Se1^i^	0.98	3.25	3.833 (3)	120
C20—H20*C*⋯Se1^ii^	0.98	3.07	3.707 (3)	124
C4—H4⋯*Cg*1^iii^	0.95	2.66	3.476 (4)	145
C15—H15⋯*Cg*1^iv^	0.95	2.90	3.699 (3)	142
C19—H19*B*⋯*Cg*2^v^	0.98	2.79	3.627 (3)	144

Symmetry codes: (i) 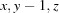; (ii) 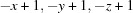; (iii) 
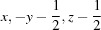
; (iv) 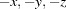; (v) 
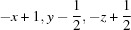
.

**Fig. 1.**
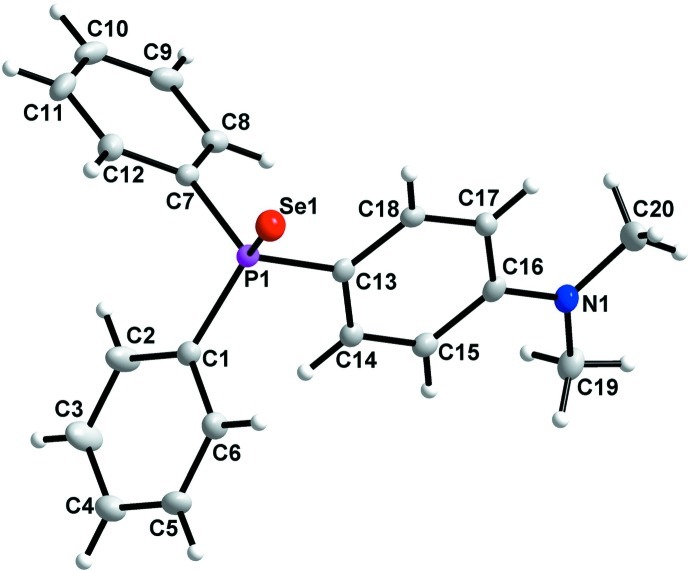
A view of the title complex, showing the atom-numbering scheme and 50% probability displacement ellipsoids.

**Fig. 2.**
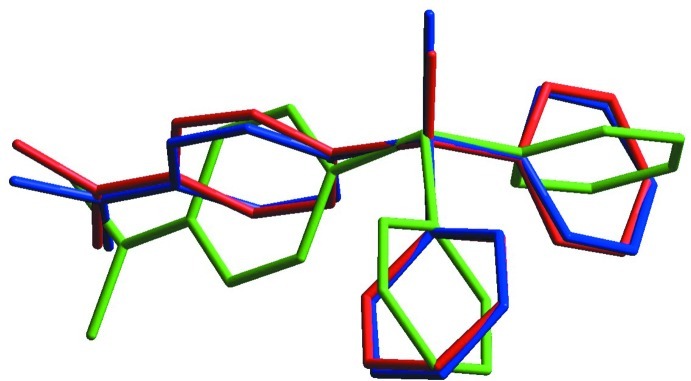
Conformational similarity between the title compound (blue), the phosphine oxide (red) and the free phosphine (green). The root mean squared deviations (RMSD) to the title compound were 0.0279 Å (oxide derivative) and 0.0473 Å (free phosphine).

**Fig. 3.**
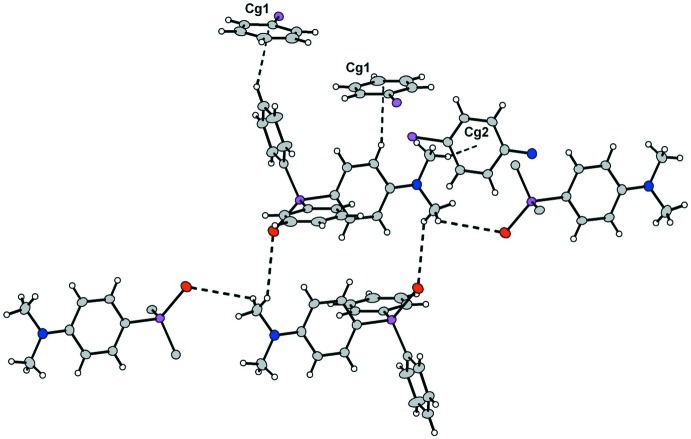
Packing diagram showing the C—H···Se/π interactions (indicated by dashed lines).

## supplementary crystallographic information

### Comment

Over the past few decades several experimental procedures to rapidly evaluate steric and electronic properties of phoshane ligands have been developed. Highlights from these studies include the measuring of IR stretching frequencies in complexes such as [NiP(CO)_3_] (Tolman, 1977), *trans*-[RhCl(CO)(P)_2_] (Roodt *et al.*, 2003; Otto & Roodt, 2004) and by the measuring of coupling constants between ^31^P and other NMR active nuclei such as ^11^B, ^195^Pt or ^77^Se (Cowley & Damasco, 1971; Allen & Taylor, 1982; Allen *et al.*, 1985). Recently our research into this area involved the use of seledized phosphane ligands, providing several useful probes such as ^1^*J*(^31^P-^77^Se) coupling, Se—P bond distance and kinetic reaction rates (Muller *et al.*, 2008) to study the steric and electronic parameters of phosphorus containing ligands. Discussed here, as part of an ongoing study, is the structure of the title compound, which is the selenium derivative of the phosphane PPh_2_(4-NMe_2_—C_6_H_4_), where Ph = C_6_H_5_.

The title compound (see Fig. 1) crystallizes in the monoclinic space group, P 2_1_/c (Z=4), with its molecules adopting a distorted tetrahedral arrangement about the phosphorus atom. The average C—P—C and Se—P—C angles are 105.28 (11)° and 113.40 (8)° respectively. The Se—P distance is 2.1069 (7) Å which is significantly shorter than the 2.1241 (5) Å reported for the analogous SePCy_2_(4-NMe_2_—C_6_H_4_) compound (Phasha *et al.*, 2012). An increase of 26 Hz in the ^1^*J*(^31^P-^77^Se) NMR coupling is also observed for the title compound compared to the dicyclohexcyl analogue. This is in accordance with Bent's rule that the s-character of the phosphorus lone pair electrons will decrease with more electron-donating substituents (Bent, 1961).

To describe the steric demand of phosphane ligands a variety of models have been developed, of which the Tolman cone angle (Tolman, 1977) is still the most commonly used method. Applying this model to the geometry obtained for the title compound (and adjusting the Se—P bond distance to 2.28 Å) we calculated an effective cone angle from the geometry found in the crystal structure as 157° (Otto, 2001). This value is comparable to the cone angles calculated for the structure of the free (Lynch *et al.*, 2003) and oxidized (Dreissig & Plieth, 1972) forms of the phosphane (calculated as 158° and 161° respectively). The orientation of the substituents for the oxidized derivative is comparable to that of the title compound, whereas the free phosphane shows substantial differences in its orientations. To illustrate this observation, the coordinates of P and *ipso* C-atoms of the three structures are superimposed using Mercury (see Fig. 2; Macrae *et al.*, 2006; Weng *et al.*, 2008*a*; Weng *et al.*, 2008*b*). The reason for the different substituent orientations are possibly due to different interactions observed to the packing of these structures. It is also interesting to note that coordination of the phosphane to transition metals does not induce significant steric crowding, and hence a smaller cone angle, of the ligand at the coordination sphere. Data extracted for these coordination complexes from the Cambridge Structural Database shows an average cone angle of 159° (Allen, 2002; 9 observations with metals: Au, Pt, Pd, Rh and Cu).

Packing in the crystals is assisted by weak C—H···Se interactions creating linked dimeric units of the title compound. In addition C—H···π interactions are also observed (see table 1 and Fig. 3 for a graphical representation of the interactions).

### Experimental

[4-(Dimethylamino)phenyl]diphenylphosphane and KSeCN were purchased from Sigma-Aldrich and used without purification. Eqimolar amounts of KSeCN (5.8 mg, 0.04 mmol) and the [4-(dimethylamino)phenyl]diphenylphosphane (12.2 mg, 0.04 mmol) were dissolved in the minimum amounts of methanol (10 ml). The KSeCN solution was added drop wise (5 min.) to the phosphane solution with stirring at room temperature. Slow evaporation of the solvent afforded the title compound as colourless crystals suitable for a single-crystal X-ray study. Analytical data: ^31^P {H} NMR (CDCl_3_, 161.99 MHz): δ = 33.62 (t, ^1^*J*(^31^P-^77^Se) = 713 Hz).

### Refinement

The aromatic and methyl H atoms were placed in geometrically idealized positions (C—H = 0.95–0.98) and allowed to ride on their parent atoms, with *U*_iso_(H) = 1.2*U*_eq_(C) for aromatic and *U*_iso_(H) = 1.5*U*_eq_(C) for methyl H atoms respectively. Methyl torsion angles were refined from electron density.

### Crystal data

**Table:**

C_20_H_20_NPSe	*F*(000) = 784
*M**_r_* = 384.3	*D*_x_ = 1.432 Mg m^−^^3^
Monoclinic, *P*2_1_/*c*	Mo *K*α radiation, λ = 0.71073 Å
Hall symbol: -P 2ybc	Cell parameters from 9940 reflections
*a* = 12.1757 (13) Å	θ = 2.3–28.3°
*b* = 10.6173 (11) Å	µ = 2.20 mm^−^^1^
*c* = 17.5211 (14) Å	*T* = 100 K
β = 128.098 (5)°	Cuboid, colourless
*V* = 1782.5 (3) Å^3^	0.22 × 0.11 × 0.09 mm
*Z* = 4	

### Data collection

**Table:**

Bruker APEX DUO 4K CCD diffractometer	4553 independent reflections
Radiation source: sealed tube	3798 reflections with *I* > 2σ(*I*)
Graphite monochromator	*R*_int_ = 0.050
Detector resolution: 8.4 pixels mm^-1^	θ_max_ = 28.7°, θ_min_ = 2.1°
φ and ω scans	*h* = −16→16
Absorption correction: multi-scan (*SADABS*; Bruker, 2008)	*k* = −14→14
*T*_min_ = 0.643, *T*_max_ = 0.827	*l* = −23→23
31924 measured reflections	

### Refinement

**Table:**

Refinement on *F*^2^	Primary atom site location: structure-invariant direct methods
Least-squares matrix: full	Secondary atom site location: difference Fourier map
*R*[*F*^2^ > 2σ(*F*^2^)] = 0.036	Hydrogen site location: inferred from neighbouring sites
*wR*(*F*^2^) = 0.097	H-atom parameters constrained
*S* = 1.06	*w* = 1/[σ^2^(*F*_o_^2^) + (0.0336*P*)^2^ + 3.5217*P*] where *P* = (*F*_o_^2^ + 2*F*_c_^2^)/3
4553 reflections	(Δ/σ)_max_ = 0.001
210 parameters	Δρ_max_ = 0.53 e Å^−^^3^
0 restraints	Δρ_min_ = −0.72 e Å^−^^3^

### Special details

**Table:**

Experimental. The intensity data was collected on a Bruker Apex DUO 4 K CCD diffractometer using an exposure time of 20 s/frame. A total of 2352 frames were collected with a frame width of 0.5° covering up to θ = 28.66° with 99.3% completeness accomplished.
Geometry. All s.u.'s (except the s.u. in the dihedral angle between two l.s. planes) are estimated using the full covariance matrix. The cell s.u.'s are taken into account individually in the estimation of s.u.'s in distances, angles and torsion angles; correlations between s.u.'s in cell parameters are only used when they are defined by crystal symmetry. An approximate (isotropic) treatment of cell s.u.'s is used for estimating s.u.'s involving l.s. planes.
Refinement. Refinement of *F*^2^ against ALL reflections. The weighted *R*-factor *wR* and goodness of fit *S* are based on *F*^2^, conventional *R*-factors *R* are based on *F*, with *F* set to zero for negative *F*^2^. The threshold expression of *F*^2^ > σ(*F*^2^) is used only for calculating *R*-factors(gt) *etc*. and is not relevant to the choice of reflections for refinement. *R*-factors based on *F*^2^ are statistically about twice as large as those based on *F*, and *R*- factors based on ALL data will be even larger.

### Fractional atomic coordinates and isotropic or equivalent isotropic displacement parameters (Å^2^)

**Table:**

	*x*	*y*	*z*	*U*_iso_*/*U*_eq_	
Se1	0.65445 (3)	0.81555 (3)	0.51517 (2)	0.02312 (9)	
P1	0.79548 (6)	0.70500 (6)	0.51220 (4)	0.01475 (13)	
N1	0.5685 (2)	0.1943 (2)	0.33365 (16)	0.0202 (4)	
C1	0.8477 (2)	0.7754 (2)	0.44440 (17)	0.0158 (5)	
C2	0.9861 (3)	0.7749 (3)	0.4794 (2)	0.0284 (6)	
H2	1.0572	0.744	0.5427	0.034*	
C3	1.0206 (3)	0.8195 (3)	0.4219 (2)	0.0354 (7)	
H3	1.1152	0.8193	0.4463	0.042*	
C4	0.9182 (3)	0.8642 (3)	0.3296 (2)	0.0236 (5)	
H4	0.9421	0.8945	0.2906	0.028*	
C5	0.7798 (3)	0.8645 (3)	0.29429 (19)	0.0222 (5)	
H5	0.7089	0.894	0.2305	0.027*	
C6	0.7448 (3)	0.8218 (3)	0.35178 (19)	0.0211 (5)	
H6	0.6504	0.8243	0.3278	0.025*	
C7	0.9584 (2)	0.6739 (2)	0.63238 (17)	0.0160 (5)	
C8	1.0104 (3)	0.5520 (2)	0.66278 (18)	0.0179 (5)	
H8	0.9605	0.4827	0.6205	0.021*	
C9	1.1364 (3)	0.5315 (3)	0.75581 (19)	0.0229 (5)	
H9	1.1713	0.4484	0.777	0.027*	
C10	1.2095 (3)	0.6329 (3)	0.81646 (19)	0.0266 (6)	
H10	1.2953	0.6191	0.8791	0.032*	
C11	1.1586 (3)	0.7546 (3)	0.78654 (19)	0.0267 (6)	
H11	1.2098	0.8235	0.8288	0.032*	
C12	1.0330 (3)	0.7762 (3)	0.69497 (19)	0.0216 (5)	
H12	0.9979	0.8595	0.6749	0.026*	
C13	0.7256 (2)	0.5533 (2)	0.45680 (17)	0.0156 (5)	
C14	0.7489 (2)	0.4999 (2)	0.39474 (17)	0.0163 (5)	
H14	0.802	0.5451	0.3809	0.02*	
C15	0.6959 (2)	0.3825 (2)	0.35318 (17)	0.0168 (5)	
H15	0.7116	0.3495	0.3102	0.02*	
C16	0.6189 (2)	0.3111 (2)	0.37367 (17)	0.0167 (5)	
C17	0.5955 (2)	0.3657 (2)	0.43629 (17)	0.0179 (5)	
H17	0.5443	0.3202	0.4517	0.022*	
C18	0.6462 (2)	0.4845 (2)	0.47526 (17)	0.0168 (5)	
H18	0.6267	0.5201	0.5155	0.02*	
C19	0.6003 (3)	0.1393 (3)	0.2728 (2)	0.0234 (5)	
H19A	0.5672	0.1957	0.2182	0.035*	
H19B	0.5536	0.0575	0.2482	0.035*	
H19C	0.7013	0.1278	0.3113	0.035*	
C20	0.5016 (3)	0.1166 (3)	0.3625 (2)	0.0278 (6)	
H20A	0.5668	0.1017	0.4326	0.042*	
H20B	0.4743	0.0359	0.3282	0.042*	
H20C	0.4184	0.1599	0.3463	0.042*	

### Atomic displacement parameters (Å^2^)

**Table:**

	*U*^11^	*U*^22^	*U*^33^	*U*^12^	*U*^13^	*U*^23^
Se1	0.02080 (14)	0.02423 (15)	0.02604 (15)	0.00213 (10)	0.01530 (12)	−0.00154 (11)
P1	0.0118 (3)	0.0177 (3)	0.0128 (3)	−0.0002 (2)	0.0066 (2)	0.0010 (2)
N1	0.0205 (10)	0.0189 (11)	0.0198 (11)	−0.0039 (8)	0.0116 (9)	−0.0033 (8)
C1	0.0143 (11)	0.0166 (11)	0.0149 (11)	0.0002 (9)	0.0081 (9)	−0.0001 (9)
C2	0.0152 (12)	0.0482 (18)	0.0205 (13)	0.0057 (12)	0.0103 (11)	0.0125 (12)
C3	0.0191 (13)	0.060 (2)	0.0305 (16)	0.0037 (14)	0.0173 (13)	0.0129 (15)
C4	0.0248 (13)	0.0277 (14)	0.0226 (13)	0.0009 (11)	0.0168 (12)	0.0028 (11)
C5	0.0216 (12)	0.0262 (13)	0.0161 (12)	0.0016 (10)	0.0102 (11)	0.0051 (10)
C6	0.0138 (11)	0.0274 (13)	0.0186 (12)	−0.0003 (10)	0.0082 (10)	0.0033 (10)
C7	0.0135 (10)	0.0210 (12)	0.0130 (11)	−0.0011 (9)	0.0078 (9)	0.0007 (9)
C8	0.0152 (11)	0.0237 (12)	0.0154 (11)	0.0014 (9)	0.0098 (10)	0.0027 (9)
C9	0.0179 (12)	0.0332 (15)	0.0192 (13)	0.0073 (10)	0.0122 (11)	0.0094 (11)
C10	0.0144 (12)	0.0492 (18)	0.0126 (12)	0.0013 (11)	0.0065 (10)	0.0035 (11)
C11	0.0200 (13)	0.0401 (17)	0.0159 (12)	−0.0093 (12)	0.0091 (11)	−0.0090 (11)
C12	0.0202 (12)	0.0248 (13)	0.0196 (13)	−0.0038 (10)	0.0121 (11)	−0.0028 (10)
C13	0.0096 (10)	0.0197 (11)	0.0124 (11)	0.0007 (9)	0.0043 (9)	0.0013 (9)
C14	0.0122 (10)	0.0192 (12)	0.0144 (11)	0.0005 (9)	0.0067 (9)	0.0010 (9)
C15	0.0138 (11)	0.0206 (12)	0.0142 (11)	0.0019 (9)	0.0077 (9)	−0.0003 (9)
C16	0.0114 (10)	0.0183 (11)	0.0126 (11)	0.0007 (9)	0.0035 (9)	0.0004 (9)
C17	0.0149 (11)	0.0221 (12)	0.0149 (11)	−0.0033 (9)	0.0082 (10)	0.0004 (9)
C18	0.0142 (11)	0.0223 (12)	0.0119 (11)	−0.0003 (9)	0.0071 (9)	0.0007 (9)
C19	0.0197 (12)	0.0226 (13)	0.0239 (13)	−0.0016 (10)	0.0114 (11)	−0.0058 (10)
C20	0.0345 (15)	0.0219 (13)	0.0248 (14)	−0.0096 (11)	0.0172 (13)	−0.0030 (11)

### Geometric parameters (Å, º)

**Table:**

Se1—P1	2.1069 (7)	C9—H9	0.95
P1—C13	1.800 (3)	C10—C11	1.388 (4)
P1—C1	1.818 (3)	C10—H10	0.95
P1—C7	1.823 (2)	C11—C12	1.392 (4)
N1—C16	1.369 (3)	C11—H11	0.95
N1—C20	1.452 (3)	C12—H12	0.95
N1—C19	1.461 (3)	C13—C18	1.399 (3)
C1—C6	1.392 (3)	C13—C14	1.402 (3)
C1—C2	1.394 (3)	C14—C15	1.386 (3)
C2—C3	1.391 (4)	C14—H14	0.95
C2—H2	0.95	C15—C16	1.414 (3)
C3—C4	1.380 (4)	C15—H15	0.95
C3—H3	0.95	C16—C17	1.417 (4)
C4—C5	1.390 (4)	C17—C18	1.385 (3)
C4—H4	0.95	C17—H17	0.95
C5—C6	1.390 (4)	C18—H18	0.95
C5—H5	0.95	C19—H19A	0.98
C6—H6	0.95	C19—H19B	0.98
C7—C8	1.394 (3)	C19—H19C	0.98
C7—C12	1.406 (4)	C20—H20A	0.98
C8—C9	1.404 (3)	C20—H20B	0.98
C8—H8	0.95	C20—H20C	0.98
C9—C10	1.383 (4)		
			
C13—P1—C1	104.77 (11)	C11—C10—H10	119.7
C13—P1—C7	106.04 (11)	C10—C11—C12	120.4 (3)
C1—P1—C7	105.02 (11)	C10—C11—H11	119.8
C13—P1—Se1	112.98 (8)	C12—C11—H11	119.8
C1—P1—Se1	113.96 (8)	C11—C12—C7	119.5 (3)
C7—P1—Se1	113.26 (8)	C11—C12—H12	120.2
C16—N1—C20	120.4 (2)	C7—C12—H12	120.2
C16—N1—C19	119.9 (2)	C18—C13—C14	117.8 (2)
C20—N1—C19	119.0 (2)	C18—C13—P1	120.45 (19)
C6—C1—C2	119.2 (2)	C14—C13—P1	121.71 (18)
C6—C1—P1	118.79 (18)	C15—C14—C13	121.4 (2)
C2—C1—P1	121.80 (19)	C15—C14—H14	119.3
C3—C2—C1	120.2 (3)	C13—C14—H14	119.3
C3—C2—H2	119.9	C14—C15—C16	121.0 (2)
C1—C2—H2	119.9	C14—C15—H15	119.5
C4—C3—C2	120.5 (3)	C16—C15—H15	119.5
C4—C3—H3	119.8	N1—C16—C15	120.9 (2)
C2—C3—H3	119.8	N1—C16—C17	121.7 (2)
C3—C4—C5	119.5 (3)	C15—C16—C17	117.3 (2)
C3—C4—H4	120.2	C18—C17—C16	120.8 (2)
C5—C4—H4	120.2	C18—C17—H17	119.6
C6—C5—C4	120.4 (2)	C16—C17—H17	119.6
C6—C5—H5	119.8	C17—C18—C13	121.6 (2)
C4—C5—H5	119.8	C17—C18—H18	119.2
C5—C6—C1	120.2 (2)	C13—C18—H18	119.2
C5—C6—H6	119.9	N1—C19—H19A	109.5
C1—C6—H6	119.9	N1—C19—H19B	109.5
C8—C7—C12	119.8 (2)	H19A—C19—H19B	109.5
C8—C7—P1	121.55 (19)	N1—C19—H19C	109.5
C12—C7—P1	118.67 (19)	H19A—C19—H19C	109.5
C7—C8—C9	120.0 (2)	H19B—C19—H19C	109.5
C7—C8—H8	120	N1—C20—H20A	109.5
C9—C8—H8	120	N1—C20—H20B	109.5
C10—C9—C8	119.7 (3)	H20A—C20—H20B	109.5
C10—C9—H9	120.1	N1—C20—H20C	109.5
C8—C9—H9	120.1	H20A—C20—H20C	109.5
C9—C10—C11	120.6 (2)	H20B—C20—H20C	109.5
C9—C10—H10	119.7		
			
C13—P1—C1—C6	74.9 (2)	C9—C10—C11—C12	0.1 (4)
C7—P1—C1—C6	−173.6 (2)	C10—C11—C12—C7	−0.6 (4)
Se1—P1—C1—C6	−49.0 (2)	C8—C7—C12—C11	0.4 (4)
C13—P1—C1—C2	−99.9 (2)	P1—C7—C12—C11	−179.1 (2)
C7—P1—C1—C2	11.6 (3)	C1—P1—C13—C18	−165.27 (19)
Se1—P1—C1—C2	136.2 (2)	C7—P1—C13—C18	84.0 (2)
C6—C1—C2—C3	−0.5 (5)	Se1—P1—C13—C18	−40.7 (2)
P1—C1—C2—C3	174.3 (3)	C1—P1—C13—C14	15.1 (2)
C1—C2—C3—C4	−0.3 (5)	C7—P1—C13—C14	−95.7 (2)
C2—C3—C4—C5	0.1 (5)	Se1—P1—C13—C14	139.70 (18)
C3—C4—C5—C6	0.9 (4)	C18—C13—C14—C15	−0.3 (3)
C4—C5—C6—C1	−1.7 (4)	P1—C13—C14—C15	179.32 (18)
C2—C1—C6—C5	1.4 (4)	C13—C14—C15—C16	−1.4 (4)
P1—C1—C6—C5	−173.5 (2)	C20—N1—C16—C15	173.9 (2)
C13—P1—C7—C8	3.9 (2)	C19—N1—C16—C15	3.2 (3)
C1—P1—C7—C8	−106.7 (2)	C20—N1—C16—C17	−6.6 (4)
Se1—P1—C7—C8	128.35 (19)	C19—N1—C16—C17	−177.3 (2)
C13—P1—C7—C12	−176.58 (19)	C14—C15—C16—N1	−179.0 (2)
C1—P1—C7—C12	72.8 (2)	C14—C15—C16—C17	1.5 (3)
Se1—P1—C7—C12	−52.1 (2)	N1—C16—C17—C18	−179.4 (2)
C12—C7—C8—C9	0.4 (4)	C15—C16—C17—C18	0.1 (3)
P1—C7—C8—C9	179.88 (19)	C16—C17—C18—C13	−1.9 (4)
C7—C8—C9—C10	−0.9 (4)	C14—C13—C18—C17	2.0 (3)
C8—C9—C10—C11	0.7 (4)	P1—C13—C18—C17	−177.68 (18)

### Hydrogen-bond geometry (Å, º)

C*g*1 and C*g*2 refer to the centroids of the C7–C12 and C13–C18 rings, respectively.

**Table:**

*D*—H···*A*	*D*—H	H···*A*	*D*···*A*	*D*—H···*A*
C20—H20*A*···Se1^i^	0.98	3.25	3.833 (3)	120
C20—H20*C*···Se1^ii^	0.98	3.07	3.707 (3)	124
C4—H4···*Cg*1^iii^	0.95	2.66	3.476 (4)	145
C15—H15···*Cg*1^iv^	0.95	2.90	3.699 (3)	142
C19—H19*B*···*Cg*2^v^	0.98	2.79	3.627 (3)	144

Symmetry codes: (i) *x*, *y*−1, *z*; (ii) −*x*+1, −*y*+1, −*z*+1; (iii) *x*, −*y*−1/2, *z*−1/2; (iv) −*x*, −*y*, −*z*; (v) −*x*+1, *y*−1/2, −*z*+1/2.
